# Supplementary material for: The Imageable Genome
Source: Nat Commun. 2023 Nov 13;14:7329. doi: 10.1038/s41467-023-43123-3 (PMC10643363; doi:10.1038/s41467-023-43123-3)
Supplement: Supplementary file 1 — Supplementary Information [file 41467_2023_43123_MOESM1_ESM.pdf]

The Imageable Genome

Supplementary Figures

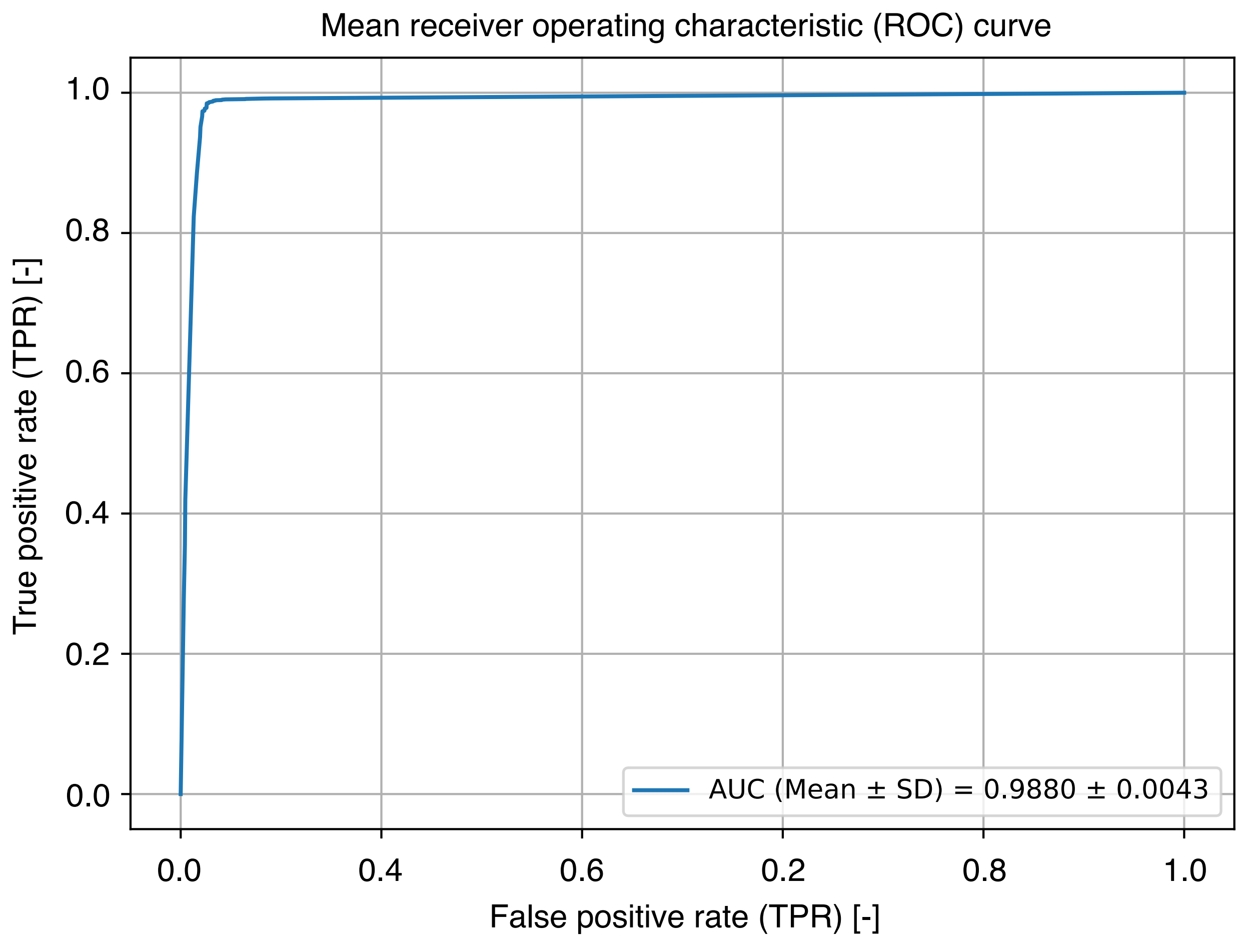

Supplementary Fig. 1. ROC curve obtained after 10 complete training processes. The AUC value is presented as Mean ± Standard Deviation. Each individual ROC curve has been built using a prediction score threshold resolution of 0.02 (from 0 to 1 in increments of 0.02) and their corresponding AUCs have been computed using numerical integration (trapezoid method) and validated using the exact methods proposed in Algorithms 1 and 2 of reference “Fawcett, Tom. (2004). ROC Graphs: Notes and Practical Considerations for Researchers. Machine Learning. 31. 1-38.”.

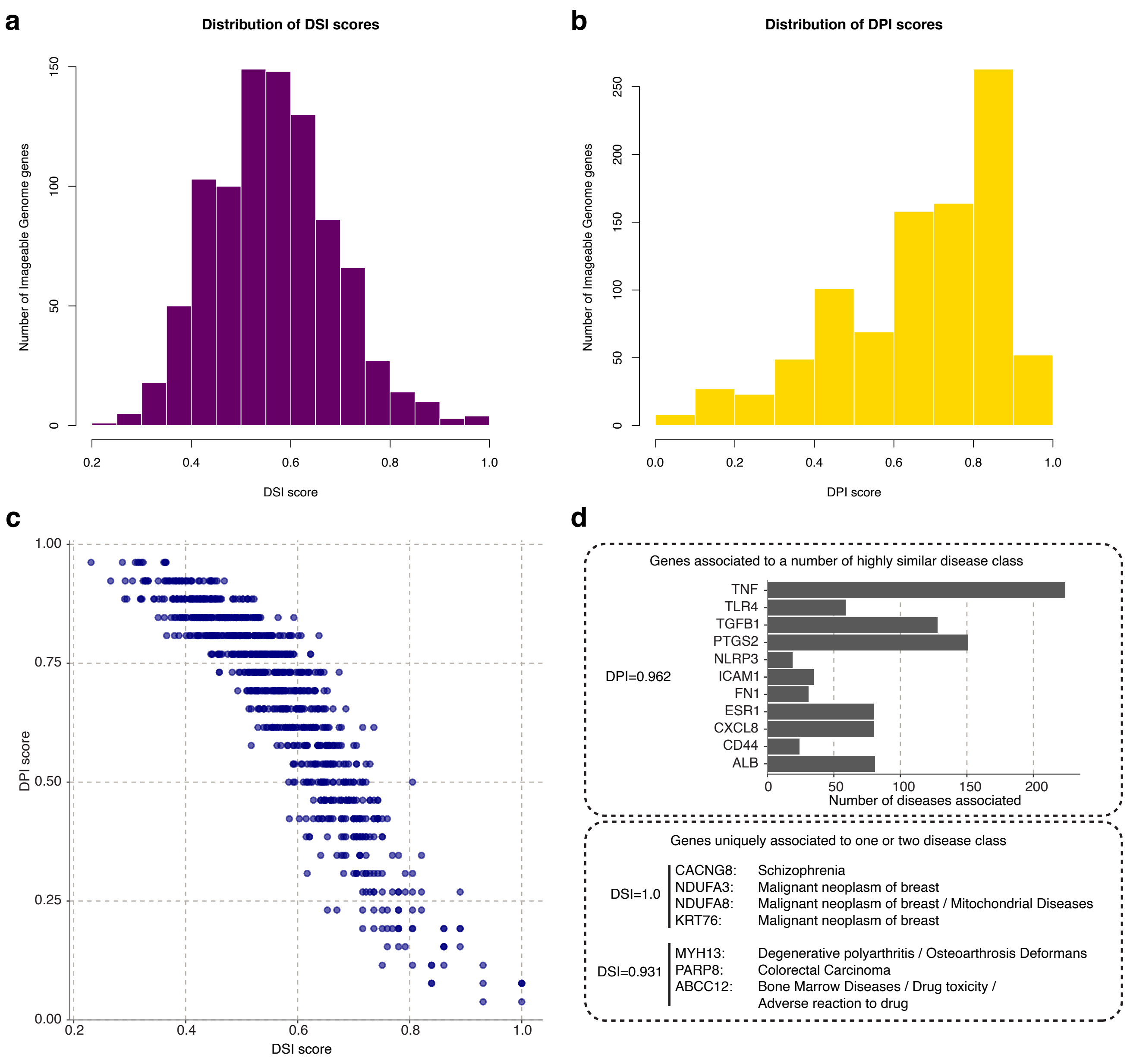

Supplementary Fig. 2. Disease Specificity index (DSI) and Disease Pleiotropy Index (DPI) distributions of Imageable Genome genes. Histograms showing the distribution of **a.** DSI Scores and **b.** DPI scores for 916 disease associated Imageable genes. **c.** A scatter plot of DSI and DPI, with top values are shown for DSI (DSI score >0.9, 7 genes) and DPI (DPI score >0.95, 11 genes), and **d.** legend to show their uniquely (or high similarly) associated disease class. Source data are provided as a Source Data file.

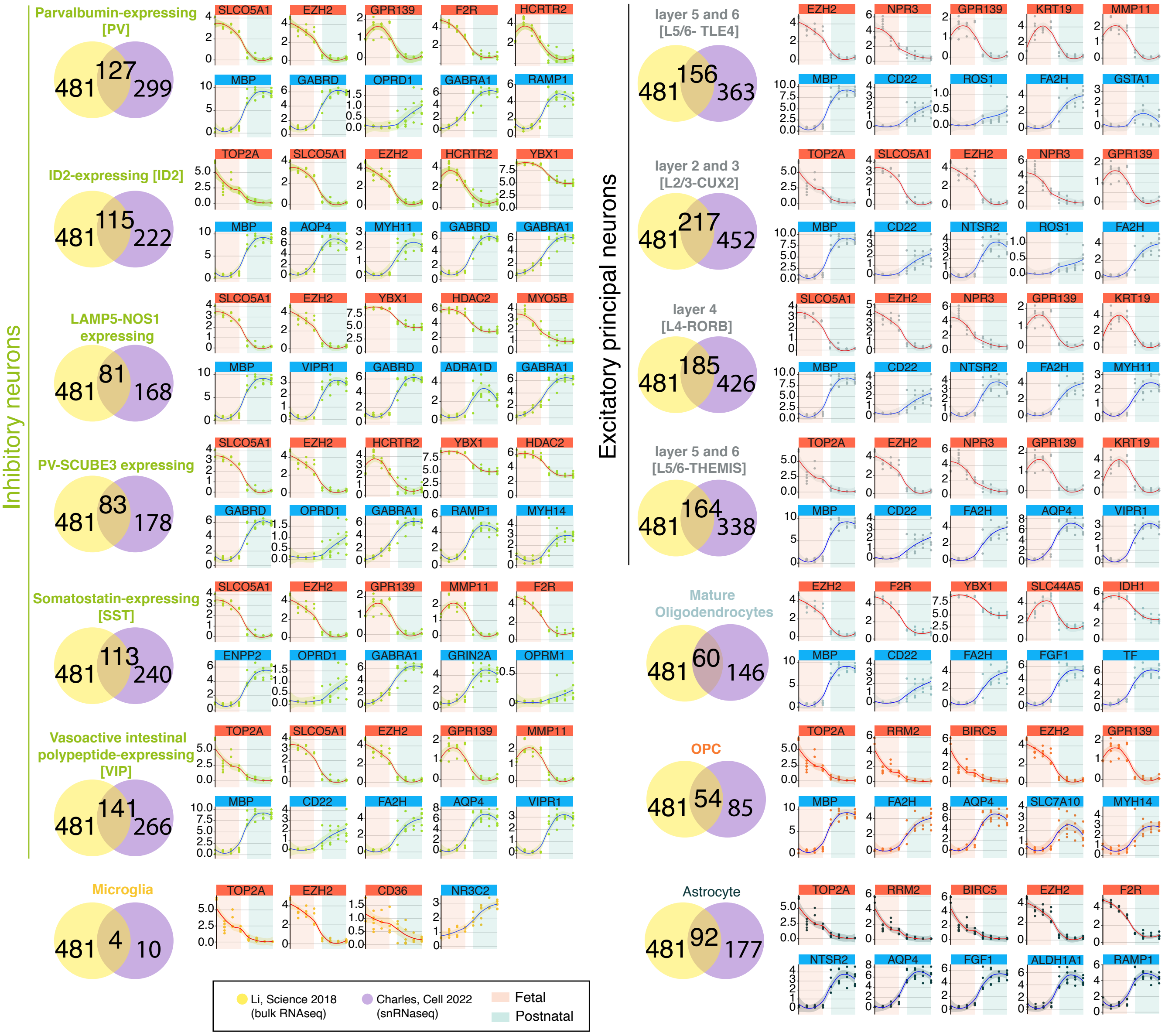

Supplementary Fig. 3. The validation set for human brain development (Charles, Cell 2022). Venn diagrams to show the number of overlapped imageable development markers identified from Li, 2018 study (bulk RNAseq data, yellow circle) and Charles, 2022 study (snRNAseq data, green circle). For each cell type, the expression patterns of top 10 genes for each cell type (top 3 genes for Mature Oligodendrocytes) that commonly upregulated in fetal stage for both studies are shown by scatter plots. The expression values are from Li, 2018. Prenatal: window 1-4 (orange box), postnatal: window 6-9 (blue box). Cell types from Charles, 2022 study: 6 major inhibitory neuron cell types, 4 major excitatory principle neuron cell types, Microglia, Oligodendrocytes precursor cells (OPC), Mature Oligodendrocytes and Astrocyte. x-axis: development window W1-4(fetal) and W6-9 (postnatal); y-axis: log2(Reads Per Kilobase per Million mapped reads+1). Source data are provided as a Source Data file.

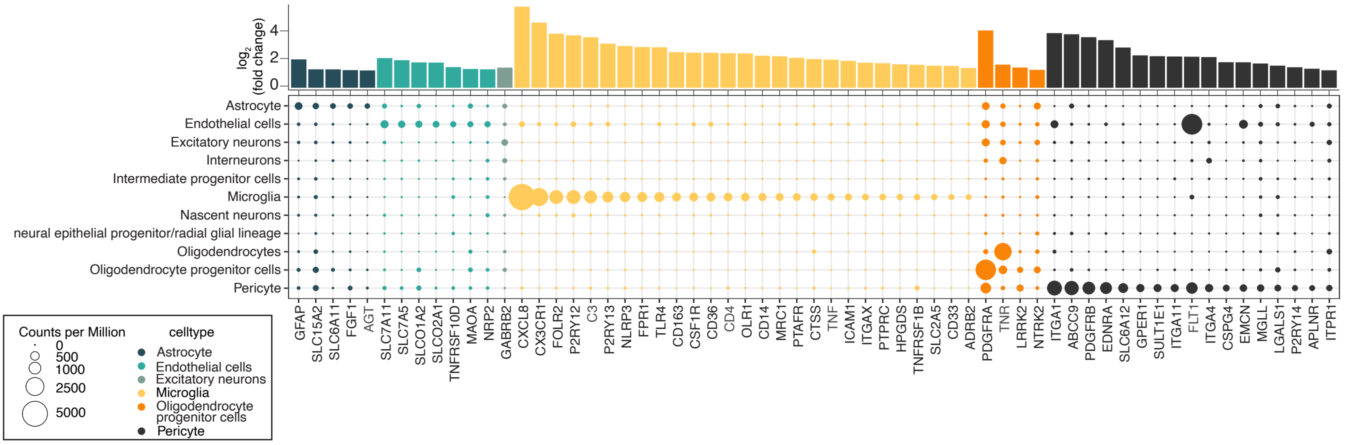

Supplementary Fig. 4. Imageable markers for embryonic brain major cell types. Dot plot depicting the expression of cell type specific imageable genes in 6 adult brain cell types, with colour code corresponding to each cell type, circle size representing the relative expression level. log2(mean expression of a gene in one cell type/ mean expression of a gene in all other cell types) are shown on top (***log2***(fold change)). Source data are provided as a Source Data file.

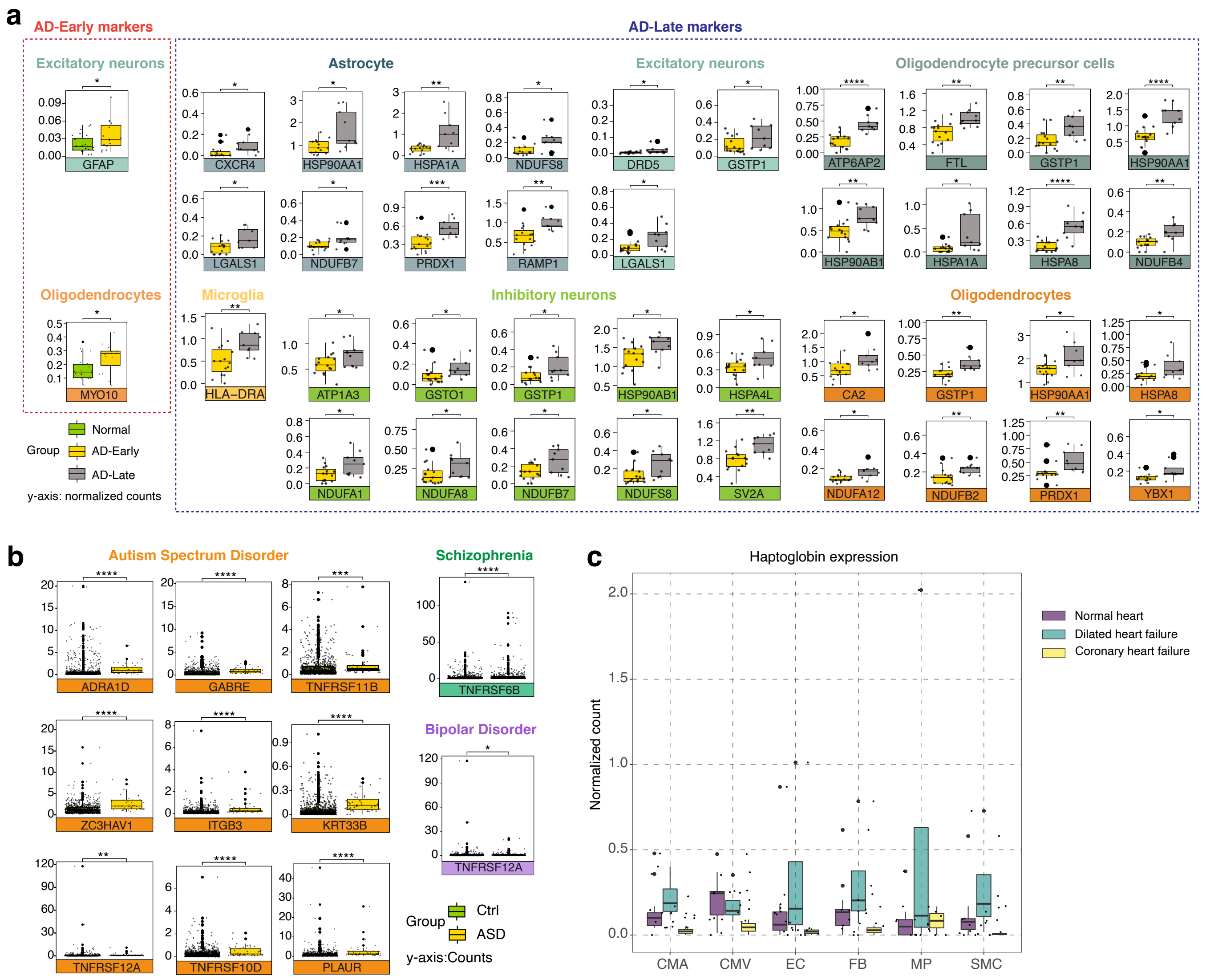

Supplementary Fig. 5. Elevated expressions of cell type specific Alzheimer's disease Imageable genes at patient level. Imageable genes with significantly elevated expression in **a**, AD-early patients (n=15) versus normal donors (n=24) or AD-Late (n=9) versus AD-early (n=15) patients and **b**, in Autism Spectrum Disorder (n=43) / Schizophrenia (n=558) / Bipolar Disorder (n=216) patients versus normal (n=921) heart donors. **c**, Haptoglobin expression in each cell type from dilated heart disease (n=4) or coronary heart disease (n=2) versus normal condition (n=14). Data are presented as mean values +/- standard deviation in (**a**-**c**), dot: individual patient, p-values by two-sided Wilcoxon rank-sum test (95% confidence interval) are shown by asterisk: *p<0.5, **p<0.01, ***p<0.001, ****p<0.0001, ns: no significance. Source data are provided as a Source Data file.

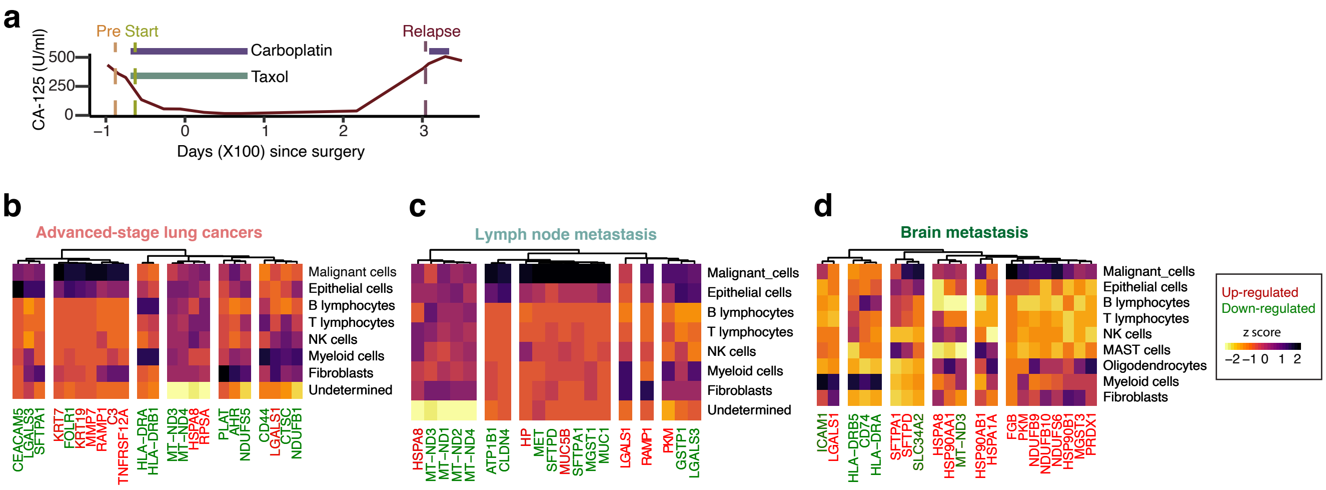

Supplementary Fig. 6. Spatiotemporal Imageable markers in human cancers. a, illustration for the disease progression and treatment window of an ovarian cancer patient used in this study. **(b,c,d)** heatmaps showing the top ranked Imageable cell type markers with significantly elevated or decreased expressions in: **b,** advanced lung cancers (n=6’400); **c,** lymph node metastasis (n=2’961); **d,** brain metastasis (n=15’423) (compared to primary tumors (n=6’352)). Source data are provided as a Source Data file.

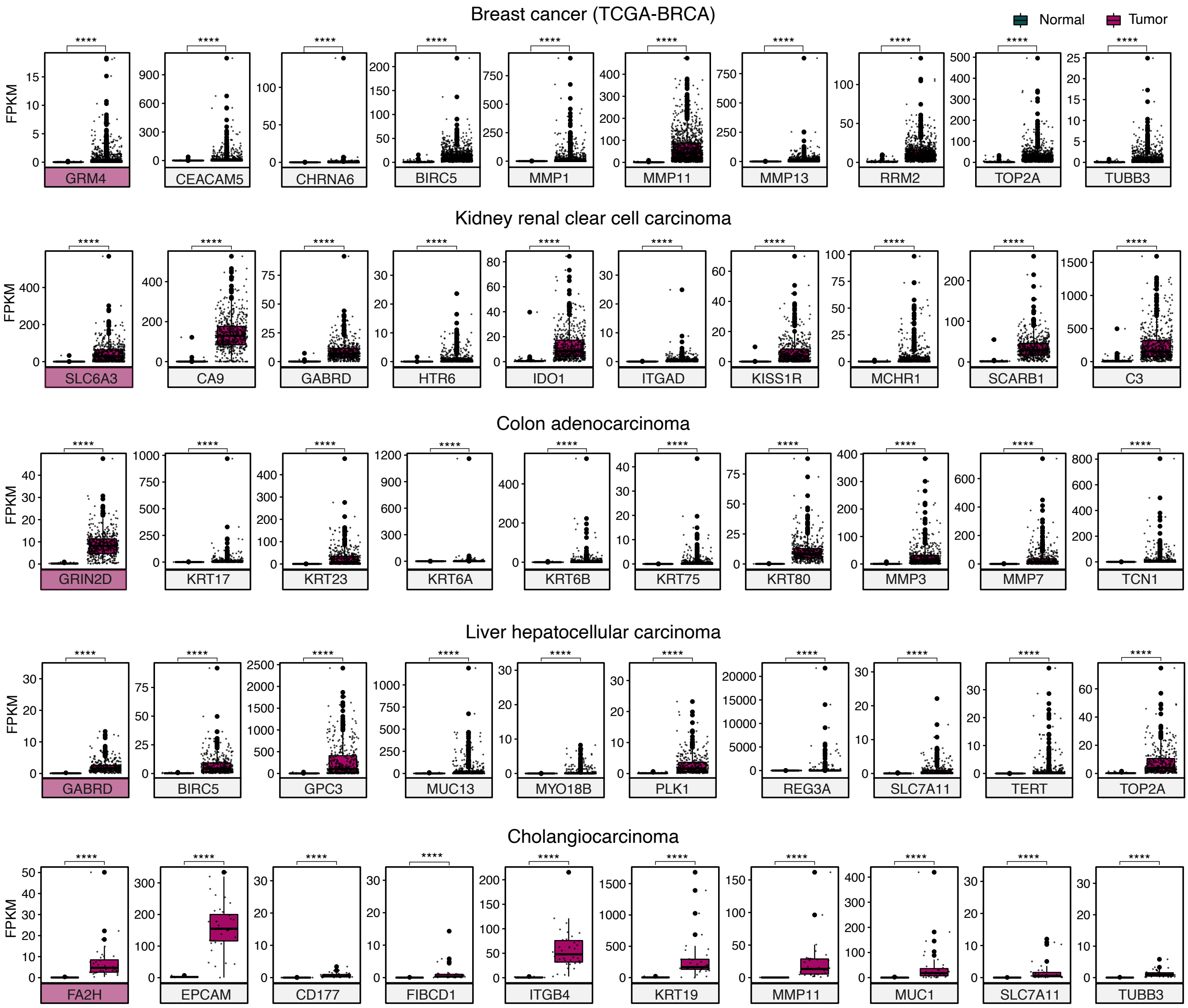

Supplementary Fig. 7. Expression of top 10 Imageable diagnostic genes across 5 TCGA cancer types at patient level. Breast cancer (n=1102) versus normal samples (n=113); Kidney renal clear cell carcinoma (n=538) versus normal samples (n=72); Colon adenocarcinoma (n=478) versus normal samples (n=41); Cholangiocarcinoma (n=36) versus normal samples (n=9). P-values by two-sided Wilcoxon rank-sum test are shown by asterisk. ****p<0.0001. Source data are provided as a Source Data file.

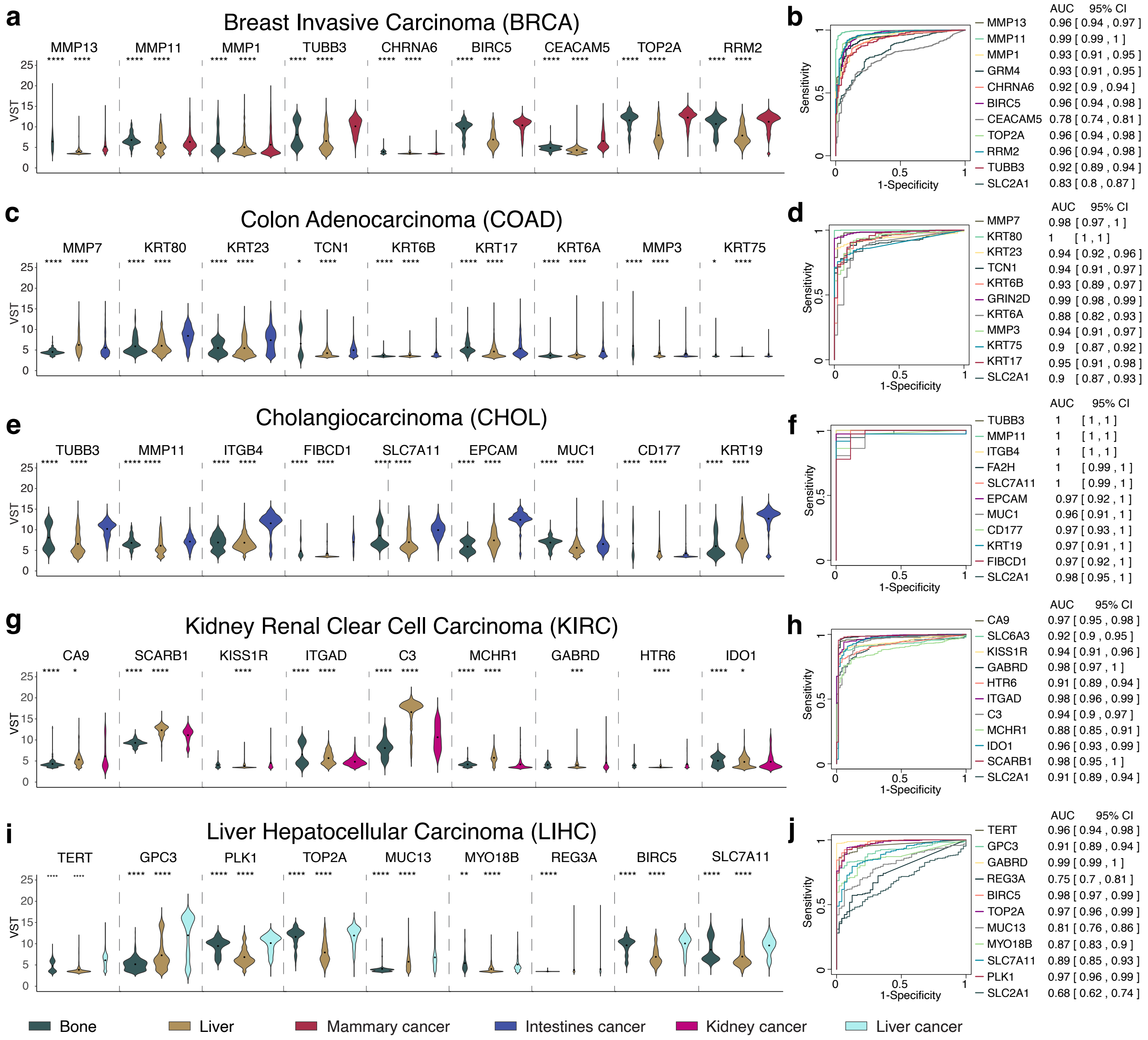

Supplementary Fig. 8. Top ranked Imageable diagnostic genes in five TCGA cancers. (a,c,e,g,i), Violin plots for the expression of top ranked 9 Imageable diagnostic genes (**Fig. 5c**) in normal bone (n=284) and liver (n=1’759) samples from GTEX database, and in cancer samples from GEO database: mammary cancer (n=5’541), kidney cancer (n=349) or intestines cancer (n =2’227). P values from two-sided Wilcoxon test are shown as asterisks. *p < 0.05; **p < 0.01; ***p<0.001; ****p<0.0001. For each tumour (TCGA samples) versus their paired normal tissue (TCGA samples), ROC curves of top ranked 10 imageable genes and GLUT1 (SLC2A1) for the diagnostic test with AUC values at 95% confidence interval (two-sided DeLong's test) are shown in **b,d,f,h,j.**

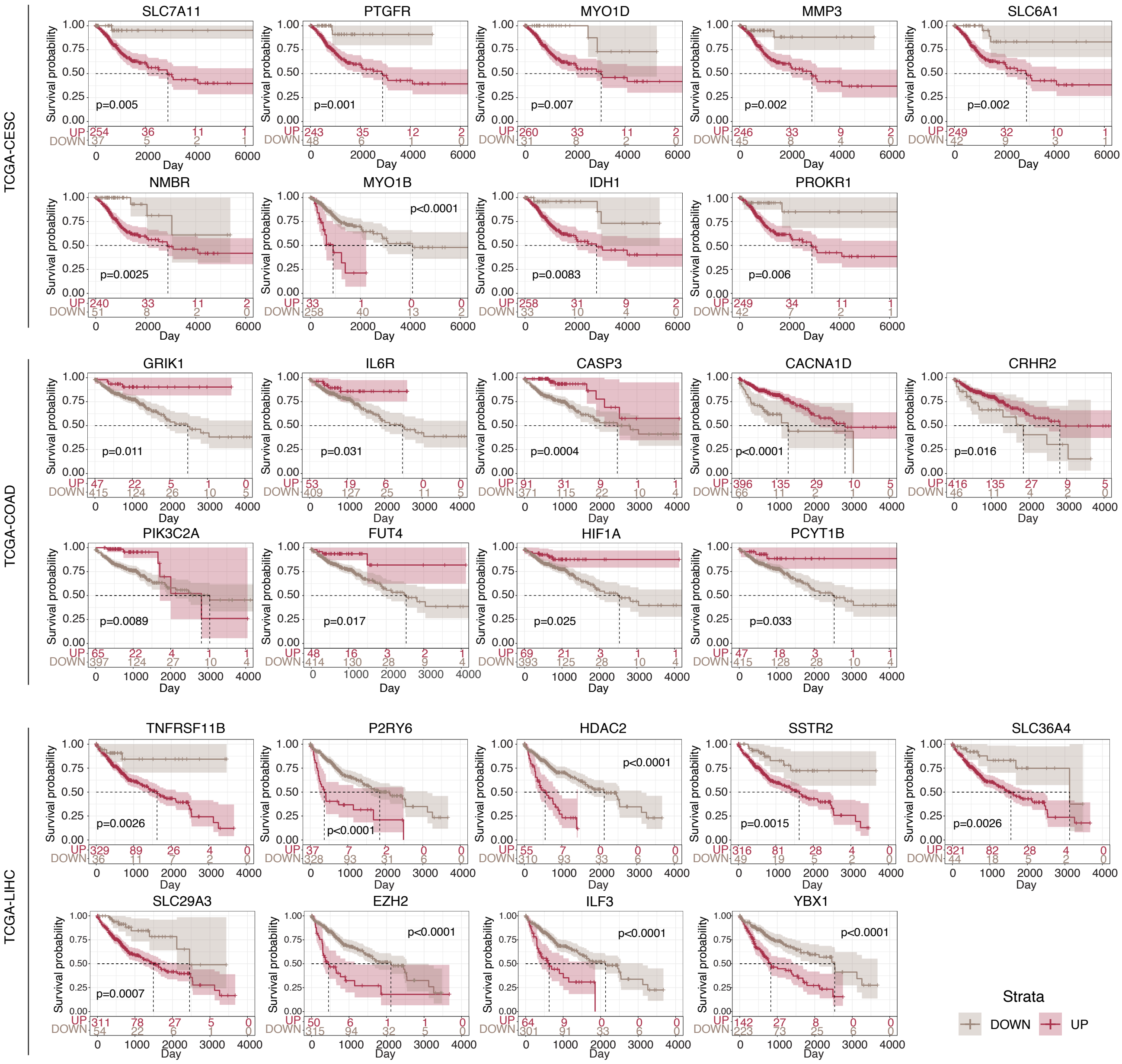

Supplementary Fig. 9. Top ranked prognostic Imageable Genome genes in three TCGA cancers. Kaplan-Meier overall survival curves (univariable hazard ratios with 95% CI and corresponding p values from Log-Rank test are shown within plots) for each top ranked Imageable Genome gene in a given TCGA cancer type.

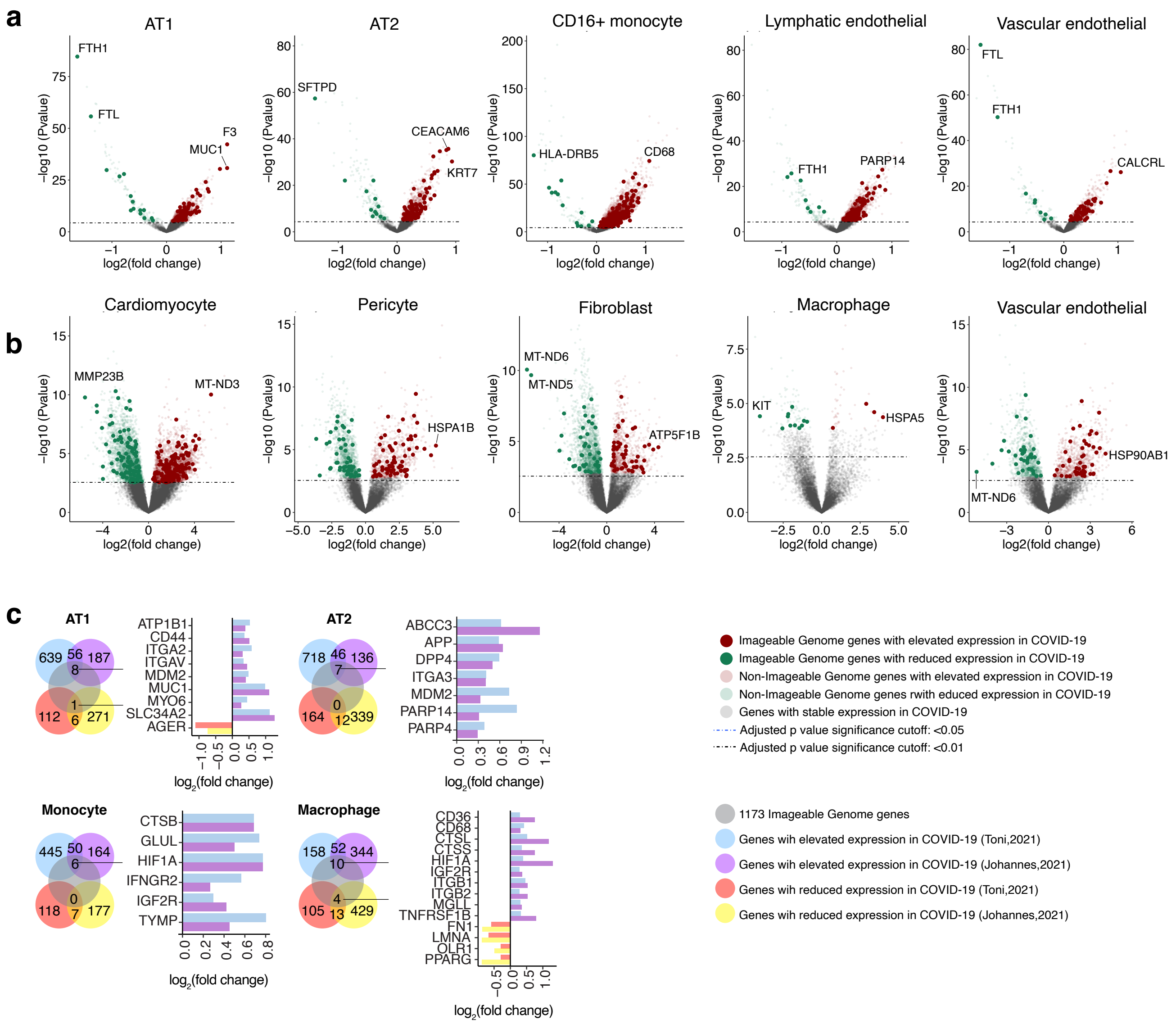

Supplementary Fig. 10. The Imageable Genome in SARS-CoV-2 infected patients. (a,b), Expression differences between COVID-19 and healthy lung and heart (Toni, 2021) shown by volcano plots of significance (-log10(Pvalue) versus magnitude (log2(fold change)) for each gene (dots) per cell type (Mann-Whitney U test, two sided). Imageable DEGs are highlighted in red and green color. Horizontal dashed line: blue, adjusted p value using a Bonferroni correction < 0.05; black, adjusted p value using a Bonferroni correction < 0.01. **c,** Venn diagrams of the number of overlapping lung cell type specific DEGs among three datasets: Toni 2021, Johannes 2021 and the Imageable Genome genes. For the imageable DEGs present in both papers, expression change of each DEG is shown by bar plot. x-axis: log2(mean expression in COVID-19 lungs /mean expression in healthy lungs), shown as: log2(fold change). Source data are provided as a Source Data file.

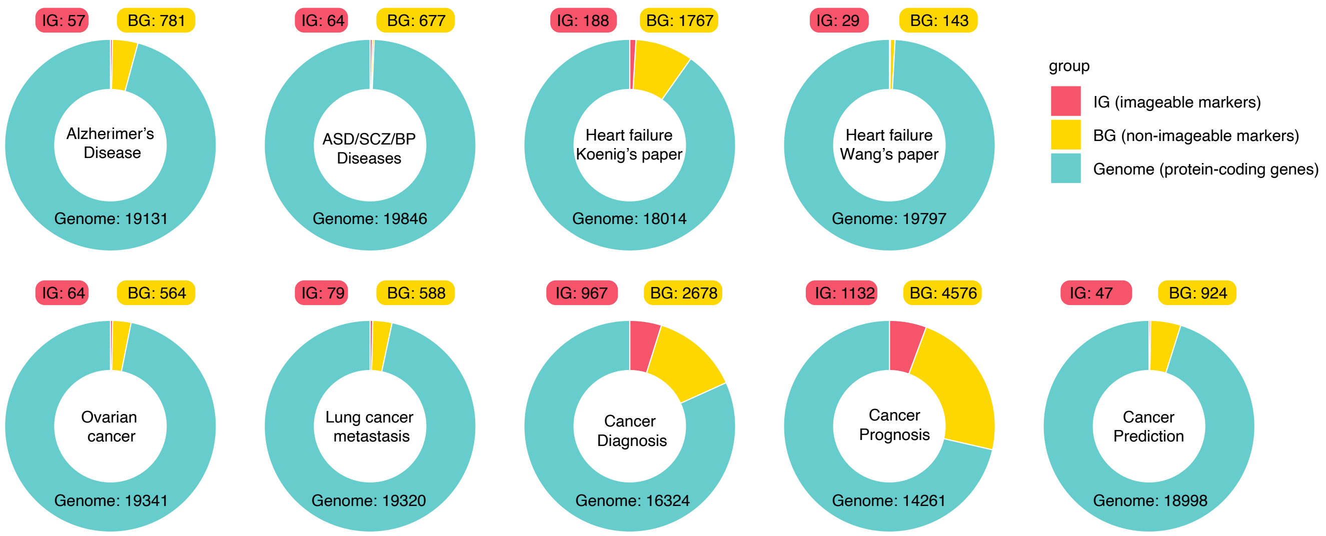

Supplementary Fig. 11. Composition of Imageable Genome genes/non-Imageable Genome genes in the complete T2T-CHM13 human genome assembly. Donut plots representing the number of unique imageable markers (red) and unique non-imageable markers (yellow) implicated in various diseases and conditions. The green part in each donut is the remaining total number of protein coding genes.
