# Supplementary material for: The Imageable Genome
Source: Nat Commun. 2023 Nov 13;14:7329. doi: 10.1038/s41467-023-43123-3 (PMC10643363; doi:10.1038/s41467-023-43123-3)
Supplement: Supplementary file 3 — Description of Additional Supplementary Files [file 41467_2023_43123_MOESM3_ESM.pdf]

Description of Additional Supplementary Files

Supplementary Data 1: The AI training datasets info.

Supplementary Data 2: The full list of radiotracer-protein-gene associations: the *Imageable Genome.*

Supplementary Data 3: The VST (Variance stabilizing transformation) value of 1161 imageable genes across 24 healthy organs.

Supplementary Data 4: The full list of imageable markers highly expressed in each brain development time window. Statistics derived from n=607 brains (n=30 window1, n=90 window2, n=54 window3, n=58 window4, n=76 window5, n=72 window6, n=72 window7, n=60 window8, n=95 window9).

Supplementary Data 5: The full list of imageable markers highly expressed in each fetal brain region. Statistics derived from n=16 fetal brain regions (n varies from 4 to 14 for each region).

Supplementary Data 6: The full list of imageable markers highly expressed in each postnatal brain region. Statistics derived from n=16 postnatal brain regions (n of each region varies from 15 to 20).

Supplementary Data 7: Adult brain imageable cell type signatures.

Supplementary Data 8: The full list of imageable markers up-/down-regulated in early-AD (n=15) versus control (n=24) or late-AD (n=9) versus early-AD, filtered at Mean_count_in>0.3 and|log2(fold change)|>0.25.

Supplementary Data 9: The full list of imageable markers up-/down-regulated in Autism (n=43), Schizophrenia (n=558), Bipolar disorder (n=216) versus normal condition (n=986).

Supplementary Data 10: The full list of un-imageable markers highly expressed in brain development time window.

Supplementary Data 11: The full list of un-imageable markers highly expressed in each fetal brain region.

Supplementary Data 12: The full list of un-imageable markers highly expressed in each postnatal brain region.

Supplementary Data 13: Adult brain cell type markers.

Supplementary Data 14: The full list of un-imageable markers up-/down-regulated in early-AD (n=15) versus control (n=24) / late-AD (n=9) versus early-AD.

Supplementary Data 15: The full list of un-imageable markers up-/down-regulated in Autism (n=43), Schizophrenia (n=558), Bipolar disorder (n=216) versus normal condition (n=986).

Supplementary Data 16: Top ranked imageable temporal markers during early embryonic heart development.

Supplementary Data 17: Full list of unique imageable regional markers of early embryonic heart.

Supplementary Data 18: Full list of regional and cell type imageable markers of adult heart.

Supplementary Data 19: Adult global imageable heart cell type markers.

Supplementary Data 20: Full list of disease/cell type-specific imageable markers in 2 human heart failure (HF) diseases. (Koenig, Nature Cardiovascular Research, 2022): 17 dilated cardiomyopathy and 28 healthy donors; (Wang, Nature Cell Biology 2020): 4 dilated cardiomyopathy / 2 coronary heart failure and 14 healthy donors.

Supplementary Data 21: Top un-imageable temporal markers during early embryonic heart development.

Supplementary Data 22: Full list of unique un-imageable regional markers of early embryonic heart.

Supplementary Data 23: Full list of regional and cell type un-imageable markers of adult heart.

Supplementary Data 24: Adult global heart un-imageable cell type markers.

Supplementary Data 25: Full list of disease/cell type-specific un-imageable markers in 3 human heart failure (HF) diseases.

Supplementary Data 26: Ovarian cancer imageable stage markers.

Supplementary Data 27: Full list of imageable markers of lung cancer metastasis. Statistics derived from pairwise comparisons between primary- (n tlung = 6352) versus advanced-stage primary (n tL/B= 6400), primary- versus metastatic cancer cells (n mLN = 2961, n mBrain = 15423, PE=396).

Supplementary Data 28: Full list of diagnostic imageable markers for 20 TCGA cancer types. For each cancer type, statistics derived from comparisons between primary solid tumor samples versus solid tissue normal samples. p values from Limma (voom()) moderate test corrected for multiple testing<0.01 and log2(fold change)>0.5.

Supplementary Data 29: Full list of imageable markers with their predictive capacity for PD1-blocakge response in ipi naïve melanoma patients. Genes are filtered at AUC>0.6 or AUC<0.35. P-values are calculated using the Wilcoxon rank-sum test with R function from package verification roc.area() (version 1.42).

Supplementary Data 30: Full list of prognostic imageable markers. The multivariable survival analyses were performed by initially computing univariable hazard ratios (95% CI) for each diagnostic IG markers that passed univariate test (p value<0.05 from two-sided Log-Rank test) with best cutoff determinded by R function surv_cutpoint() (Package" survminer"); followed by fitting Cox proportional hazards models (CPH) with best cutoff, filtered at adjust p value <0.05 (corrected for multiple testing on p vlaues from Wald statistic) & HR>1.5 | HR<0.7.

Supplementary Data 31: Ovarian cancer un-imageable stage markers. P values from two-sided Wilcoxon test. Log2FoldChange calculated average expression of gene in tested group relative to the average expression in reference group.

Supplementary Data 32: Full list of un-imageable markers of lung cancer metastasis. Statistics derived from pairwise comparisons between primary- (n tlung = 6352) versus advanced-stage primary (n tL/B= 6400), or primary- versus metastatic cancer cells (n mLN = 2961, n mBrain = 15423).

Supplementary Data 33: Full list of diagnostic un-imageable markers for 23 TCGA cancer types. For each cancer type, statistics derived from comparisons between primary solid tumor samples versus solid tissue normal samples. p values from Limma moderate test corrected for multiple testing<0.001 and log2(fold change)>5.

Supplementary Data 34: Full list of un-imageable markers with their predictive capacity for PD1-blocakge response in ipi naïve melanoma patients. Genes are filtered at AUC>0.6 or AUC<0.35.

Supplementary Data 35: Full list of prognostic un-imageable markers. Top background DEGs passed univariate test (log2(fold change)>0.5 & p value<0.05 from two-sided Log-Rank test), and multivariate test with best cutoff, filtered at adj.p<0.001(corrected for multiple testing on p values from Wald statistic) & HR>1.5 | HR<0.7.

Supplementary Data 36: Top 10 clinical frequently used PET radiotracers and their clinical applications.

Supplementary Data 37: The full list of prognostic capacity of top 10 clinical frequently used PET radiotracers.
