# Supplementary material for: The Imageable Genome
Source: Nat Commun. 2023 Nov 13;14:7329. doi: 10.1038/s41467-023-43123-3 (PMC10643363; doi:10.1038/s41467-023-43123-3)
Supplement: Supplementary file 5 — Reporting Summary [file 41467_2023_43123_MOESM5_ESM.pdf]

nature portfolio

Corresponding author(s): Pablo Jané, Xiaoying Xu, Vincent Taelman, Eduardo Jané , Karim Gariani , Rebecca A. Dumont , Yonathan Garama,, Francisco Kim, María del Val Gomez, and Martin A. Walter

Last updated by author(s): 19/10/23

Reporting Summary

Nature Portfolio wishes to improve the reproducibility of the work that we publish. This form provides structure for consistency and transparency in reporting. For further information on Nature Portfolio policies, see our Editorial Policies and the Editorial Policy Checklist.

Statistics

For all statistical analyses, confirm that the following items are present in the figure legend, table legend, main text, or Methods section.

n/a	Confirmed	
☐	☒	The exact sample size (*n*) for each experimental group/condition, given as a discrete number and unit of measurement
☐	☒	A statement on whether measurements were taken from distinct samples or whether the same sample was measured repeatedly
☐	☒	The statistical test(s) used AND whether they are one- or two-sided<br>*Only common tests should be described solely by name; describe more complex techniques in the Methods section.*
☐	☒	A description of all covariates tested
☐	☒	A description of any assumptions or corrections, such as tests of normality and adjustment for multiple comparisons
☐	☒	A full description of the statistical parameters including central tendency (e.g. means) or other basic estimates (e.g. regression coefficient) AND variation (e.g. standard deviation) or associated estimates of uncertainty (e.g. confidence intervals)
☐	☒	For null hypothesis testing, the test statistic (e.g. F, t, r) with confidence intervals, effect sizes, degrees of freedom and P value noted<br>*Give P values as exact values whenever suitable.*
☒	☐	For Bayesian analysis, information on the choice of priors and Markov chain Monte Carlo settings
☒	☐	For hierarchical and complex designs, identification of the appropriate level for tests and full reporting of outcomes
☒	☐	Estimates of effect sizes (e.g. Cohen's *d*, Pearson's *r*), indicating how they were calculated

Our web collection on statistics for biologists contains articles on many of the points above.

Software and code

Policy information about availability of computer code

Data collection	The imagenome package contains all the necessary tools to reproduce the data pipeline of the Imageable Genome project. (GNU Affero General Public License v3.0)<br>Code available at : https://github.com/pablojane/ImageableGenome
Data analysis	The imagenome package contains all the necessary tools to reproduce the data pipeline of the Imageable Genome project. (GNU Affero General Public License v3.0)<br>Code available at : https://github.com/pablojane/ImageableGenome

For manuscripts utilizing custom algorithms or software that are central to the research but not yet described in published literature, software must be made available to editors and reviewers. We strongly encourage code deposition in a community repository (e.g. GitHub). See the Nature Portfolio guidelines for submitting code & software for further information.

Data

Policy information about availability of data

All manuscripts must include a data availability statement. This statement should provide the following information, where applicable:

- Accession codes, unique identifiers, or web links for publicly available datasets
- A description of any restrictions on data availability
- For clinical datasets or third party data, please ensure that the statement adheres to our policy

The data generated in this study are provided in the Supplementary Information and Source Data file. Source data are provided with this paper.

All raw data used in this study are publicly available and can be found as follows:
Sc/snRNA-seq data from the human brain development study (Li, Science 2018) are available at PsychENCODE Knowledge Portal with Project SynID: syn4921369, under DOI https://doi.org/10.7303/syn4921369. (SynapseID: syn17092080 and syn17092080). Count matrix and annotations have been deposited at http://psychencode.org. SnRNA-seq for Human brain development (Charles, Cell 2022) is available in the Expression Omnibus (GEO) database under accession code: GSE168408 (https://www.ncbi.nlm.nih.gov/geo/query/acc.cgi?acc=GSE168408). Bulk RNA-seq data from the ASD, SCZ and BP brain disease study (Gandal, Science 2018) are available at PsychENCODE Knowledge Portal with SynapseID: syn12080241 under DOI https://doi.org/10.7303/syn12080241. SnRNA-seq data from the Alzheimer's disease study (Mathys, Nature 2019) are available at AD Knowledge Portal with SynapseID: syn18485175 under the DOI https://doi.org/10.7303/syn18485175. The raw sequencing data from human embryonic heart development study (Asp, Cell 2019) is under European Genome-phenome Archive (EGA) accession number: EGAS0000100399. Count matrices and annotation are available at https://www.spatialresearch.org.
The raw sequencing data from adult human heart cell atlas (Litviňuková, Nature 2020) are available at the Human Cell Atlas (HCA) Data Coordination Platform (DCP) with accession number: ERP123138. Count matrices and annotation are available for download from the Heart Cell Atlas (https://www.heartcellatlas.org). ScRNA-seq data from dilated cardiomyopathy (dHF) or coronary heart disease study (Wang, Nature Cell Biology 2020) are available in the GEO database under accession code: GSE109816 (https://www.ncbi.nlm.nih.gov/geo/query/acc.cgi?acc=GSE109816) and GSE121893 (https://www.ncbi.nlm.nih.gov/geo/query/acc.cgi?acc=GSE121893). Sc/snRNA-seq from dilated cardiomyopathy (dHF) study (Koenig, Nature Cardiovascular Research, 2022) are available in the GEO database under accession code: GSE183852 (https://www.ncbi.nlm.nih.gov/geo/query/acc.cgi?acc=GSE183852). Bulk RNA-seq data from human atrial fibrillation study (van Ouwerkerk, Nat Communications, 2019) are available in the GEO database under accession code: GSE127856 (https://www.ncbi.nlm.nih.gov/geo/query/acc.cgi?acc=GSE127856).
ScRNA-seq data from Ovarian cancer study (Nath, Nat Commun 2021) are available in the GEO database under the accession code: GSE158722 (https://www.ncbi.nlm.nih.gov/geo/query/acc.cgi?acc=GSE158722). ScRNA-seq data from Lung cancer metastasis study (Kim, Nat Commun 2020) are available under GEO accession code: GSE131907 (https://www.ncbi.nlm.nih.gov/geo/query/acc.cgi?acc=GSE131907).
Sc/snRNA-Seq and bulk RNA-seq data from COVID-19 study (Toni, Nature 2021) are available in the GEO database under GEO accession code: GSE171668 (https://www.ncbi.nlm.nih.gov/geo/query/acc.cgi?acc=GSE171668). ScRNA-seq data from COVID-19 study (Johannes, Nature 2021) are available in the GEO database under accession code: GSE171524 (https://www.ncbi.nlm.nih.gov/geo/query/acc.cgi?acc=GSE171524), and in the single-cell portal: https://singlecell.broadinstitute.org/single_cell/study/SCP1219.

Research involving human participants, their data, or biological material

Policy information about studies with human participants or human data. See also policy information about sex, gender (identity/presentation), and sexual orientation and race, ethnicity and racism.

Reporting on sex and gender	n/a
Reporting on race, ethnicity, or other socially relevant groupings	n/a
Population characteristics	n/a
Recruitment	n/a
Ethics oversight	n/a

Note that full information on the approval of the study protocol must also be provided in the manuscript.

Field-specific reporting

Please select the one below that is the best fit for your research. If you are not sure, read the appropriate sections before making your selection.

☒ Life sciences ☐ Behavioural & social sciences ☐ Ecological, evolutionary & environmental sciences

For a reference copy of the document with all sections, see nature.com/documents/nr-reporting-summary-flat.pdf

Life sciences study design

All studies must disclose on these points even when the disclosure is negative.

Sample size	All data used in this study are previously published datasets. We used sample size ranged from n=2 to n>1000 to address biological questions

Sample size	and significant statistical results. The increase of sample size in future study will help to exam the current findings and explore other significant findings.
Data exclusions	no data exclusion in this study.
Replication	validation datasets were used for the reproduction of findings.
Randomization	no randomization.
Blinding	no blinding.

Reporting for specific materials, systems and methods

We require information from authors about some types of materials, experimental systems and methods used in many studies. Here, indicate whether each material, system or method listed is relevant to your study. If you are not sure if a list item applies to your research, read the appropriate section before selecting a response.

Materials & experimental systems

n/a	Involved in the study	
☒	☐	Antibodies
☒	☐	Eukaryotic cell lines
☒	☐	Palaeontology and archaeology
☒	☐	Animals and other organisms
☐	☒	Clinical data
☒	☐	Dual use research of concern
☒	☐	Plants

Methods

n/a	Involved in the study	
☒	☐	ChIP-seq
☒	☐	Flow cytometry
☒	☐	MRI-based neuroimaging

Clinical data

Policy information about clinical studies

All manuscripts should comply with the ICMJE guidelines for publication of clinical research and a completed CONSORT checklist must be included with all submissions.

Clinical trial registration	n/a
Study protocol	n/a
Data collection	n/a
Outcomes	n/a
